# Exosomal miR-628-5p from M1 polarized macrophages hinders m6A modification of circFUT8 to suppress hepatocellular carcinoma progression

**DOI:** 10.1186/s11658-022-00406-9

**Published:** 2022-12-06

**Authors:** Liyan Wang, Xiaoyuan Yi, Xuhua Xiao, Qinghua Zheng, Lei Ma, Bin Li

**Affiliations:** Digestive Department, Affiliated Hospital of Guilin Medical College, No.15 Lequn Road, Xiufeng District, Guilin, 541001 Guangxi China

**Keywords:** Hepatocellular carcinoma, Exosomes, M1 macrophages, circFUT8, m6A modification

## Abstract

**Background:**

Hepatocellular carcinoma (HCC) is the most common type of liver cancer. CircFUT8 has been shown to be upregulated in cancers, but its function in HCC remains unclear. Tumor-associated macrophages (TAMs) are one of the main components of the tumor microenvironment (TME), and M1 macrophages function as tumor suppressors in cancers. Exosomes exert an important role in the TME, and circRNAs can be modified by m6A. We investigated the function of circFUT8 in HCC and its interaction with exosomes, M1 macrophages, and m6A.

**Methods:**

CircFUT8 expression was detected in HCC cells, and its effects on HCC cell growth were verified through functional assays. Mechanism assays including RNA pull down, RNA-binding protein immunoprecipitation (RIP), and luciferase reporter assays were undertaken to verify how circFUT8 may interact with miR-628-5p, and how these molecules may modulate HCC cell malignancy via interacting with exosomes and macrophages.

**Results:**

CircFUT8 was upregulated in HCC cells and it accelerated HCC cell growth. Exosomes derived from M1 macrophages transferred miR-628-5p to HCC cells to inhibit human methyltransferase-like 14 (METTL14) expression. METTL14 promoted circFUT8 m6A modification and facilitated its nuclear export to the cytoplasm, where M1 macrophages regulated the circFUT8/miR-552-3p/CHMP4B pathway, thereby suppressing HCC progression.

**Conclusion:**

M1 macrophages-derived exosomal miR-628-5p inhibited the m6A modification of circFUT8, inhibiting HCC development.

**Graphical Abstract:**

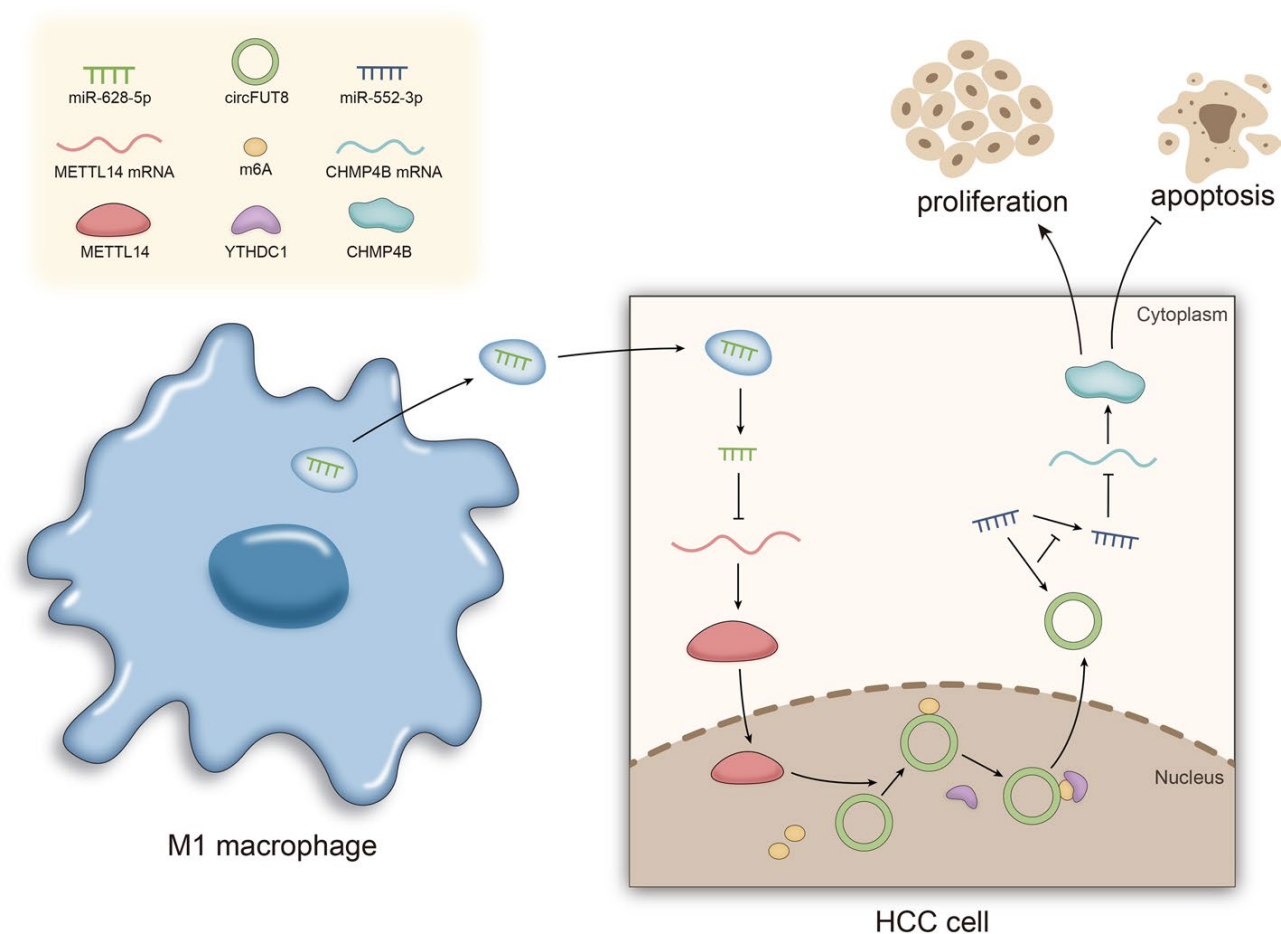

**Supplementary Information:**

The online version contains supplementary material available at 10.1186/s11658-022-00406-9.

## Background

As the most common primary liver cancer, hepatocellular carcinoma (HCC) is becoming a great risk for human health, with its characteristics of high morbidity and mortality, and the incidence of HCC and HCC-related deaths have increased over the last several decades [1]. Though advances have been made in medical and surgical therapies for HCC, the treatment and prognosis of patients with HCC are still poor, hence having a deep understanding of HCC may help discover more effective molecular targets for HCC.

Circular RNAs (circRNAs), endogenous noncoding RNAs predominantly generated by back splicing [2], are characterized by a closed circular structure, making them more stable than linear RNAs. In recent decades, more and more circRNAs have been shown to regulate the malignant development of cancers: circRNA_0000285 aggravates the development of cervical cancer via FUS [3], while circRNA WHSC1 accelerates endometrial cancer development through the miR-646/NPM1 axis [4]. Importantly, circRNAs exerting regulatory functions in the regulation of HCC has been discussed in many studies. For example, circZFR aggravates HCC cell malignancy by targeting miR-511 and AKT1 [5]. CircRNA-104718 functions as the sponge for miRNA-218-5p to modulate TXNDC5, thereby facilitating HCC progression [6]. CircRNA-5692, together with miR-328-5p and DAB2IP constitutes a regulatory pathway that attenuates the development of HCC [7]. As a novel circRNA, circFUT8 has been illustrated to be differentially expressed in cancer cells, having high expression in liver cancer while being downregulated in breast cancer [8, 9]; but how it may function in HCC has not yet been determined.

As one of the main components of the tumor microenvironment, tumor-associated macrophages (TAMs) are involved in the malignant progression of cancers [10]. Macrophages are involved in several processes in physiological and pathological conditions, and there are two major polarization states mainly implicated for macrophages, namely the activated type 1 (M1) and the alternatively activated type 2 (M2) macrophages [11]. Functionally speaking, M1 macrophages are known as tumor suppressors, while M2 macrophages exert tumor-promoting roles in cancers. MicroRNAs (miRNAs) can modulate macrophage polarization, affecting the development of HCC [12, 13]. However, the detailed mechanism concerning macrophages still needs to be further explored.

Exosomes are an important medium of communication between cancer and noncancer cells in the tumor microenvironment, and exosomes derived from cancer cells are capable of modifying local as well as distant microenvironments [14]. Moreover, tumor-derived exosomes have been implicated in the formation and progression of cancer processes, such as invasion, metastasis, and drug resistance [15]. Exosomal long noncoding RNA (lncRNA) TUG1 from cancer-associated fibroblasts promotes liver cancer cell migration, invasion, and glycolysis by regulating the miR-524-5p/SIX1 axis [16].

Representing the most abundant internal modification of RNA in eukaryotic cells, N6-methyladenosine (m6A) impacts multiple aspects of RNA metabolism, from RNA processing, nuclear export, and RNA translation to decay [17]. Related reports have identified the relationship between circRNAs and m6A modification [18]. m6A modification of circNSUN2 modulates cytoplasmic export and then stabilizes HMGA2 in the acceleration of colorectal liver metastasis [19]. Therefore, we have also tried to verify the relationship between circFUT8 and m6A modification in HCC cells.

## Methods

### Cell culture

HCC cell lines (Huh7, HCCLM3, Hep3B, MHCC97H), immortalized human liver epithelial THLE-3 cell line, and macrophage THP-1 were all obtained from ATCC (Manassas, VA, USA). The THLE-3 cell line and four HCC cell lines were cultured in RPMI-1640 medium (A4192301, Gibco, Grand Island, New York, USA) with 10% fetal bovine serum (FBS; no. 12483020, Gibco, Grand Island, New York, USA), and 1% penicillin–streptomycin (no. 15070063, Gibco, Grand Island, New York, USA). The THP-1 cell line was cultivated in RPMI-1640 medium containing 10% heat-inactivated FBS, 10 mM Hepes (no. 15630-056, Gibco, Grand Island, New York, USA), 1 mM pyruvate (#11360-039, Gibco, Grand Island, New York, USA), 2.5 g/L D-glucose, and 50 pM β-mercaptoethanol (no. 31350-010, Gibco, Grand Island, New York, USA). Cells were cultured in a humid incubator with 5% CO_2_ at 37 °C.

### Macrophage polarization model

The macrophage polarization model was constructed as previously described [11]. THP-1 cells were differentiated into M0 macrophages by 24 h incubation with 150 nM phorbol 12-myristate 13-acetate (PMA; P8139, Sigma-Aldrich, St. Louis, Missouri, USA) followed by 24 h incubation in RPMI medium. M0 macrophages were polarized in M1 macrophages by incubation with 20 ng/ml of IFN-γ (no. 285-IF, R&D system, Minneapolis, Minnesota, USA) and 10 pg/ml of lipopolysaccharide (LPS; no. 8630, Sigma-Aldrich, St. Louis, Missouri, USA).

### Cell transfection

For the overexpression of circFUT8, METTL14, and CHMP4B, the whole sequences were separately synthesized and subcloned into pcDNA3.1 vectors with a pcDNA3.1 empty vector (V79520, Invitrogen, Carlsbad, CA, USA) as a negative control (NC), while for the overexpression of miR-552-3p and miR-628-5p, related miRNA mimics and NCs were used. For the knockdown of circFUT8, METTL14, and CHMP4B, specific short hairpin RNAs (shRNAs) were respectively designed and established with nontargeting shRNA (sh-NC), while miR-552-3p/miR-628-5p inhibitors were used to silence miR-552-3p/miR-628-5p expression. In line with the supplier’s protocols, transfections were conducted with Lipofectamine 2000 (Invitrogen).

### Quantitative real-time RT–PCR (RT–qPCR)

Based on the user’ guidance for Trizol reagent (15,596,018, Invitrogen, Carlsbad, CA, USA), total RNA was extracted from HCC cells and then reverse transcribed into cDNA using SuperScript III First-Strand Synthesis SuperMix (11,752,050, Invitrogen, Carlsbad, CA, USA). The RT–qPCR reaction was achieved with the SYBR Green PCR Master Mix (4,364,346, Applied Biosystems, Foster City, CA, USA) and gene expression was calculated by the 2^−ΔΔCt^ method. In relevant assays, U6 and GAPDH served as the endogenous controls.

### Colony formation assay

Huh7 and MHCC97H cells were seeded into 6-well plates for 2 weeks of incubation. The culture medium was discarded and cells were washed with phosphate-buffered saline (PBS) twice. Methanol solution was applied for cell fixation for 15 min and crystal violet was utilized for cell staining for 10 min at room temperature. Finally, colonies with no less than 50 cells were manually counted.

### 5-Ethynyl-20-deoxyuridine (EdU) incorporation assay

Using BeyoClick EdU-488 kit (C0071S, Beyotime, Shanghai, China), HCC cells at the logarithmic growth stage were taken and seeded in 96-well plates to the normal growth stage. An appropriate amount of 50 μM EdU culture medium was prepared by dilution of EdU solution by 1:1000. Each well was added with 100 μL 50 μM EdU assay kit for cultivation for 2 h, and then the culture medium was discarded. PBS was used to wash the cells for 5 min, and 4′,6-diamidino-2-phenylindole (DAPI) was added to stain the nucleus for 5 min at room temperature. Finally, cell proliferation was monitored under fluorescence microscopy.

### Western blot

Total protein extracted from HCC cell lines was isolated by Radio Immunoprecipitation Assay (RIPA) buffer, and after being separated through Sodium dodecyl sulfate-polyacrylamide gel electrophoresis (SDS-PAGE), proteins were transferred to polyvinylidene difluoride (PVDF) membranes and blocked in 5% skim milk. The membranes were cultivated with primary antibodies against CD63 (ab134045, Abcam, Cambridge, MA, USA) and CD81 (ab109201, Abcam, Cambridge, MA, USA) overnight at 4 °C, followed by cultivation with secondary antibody for 1 h. After washing in TBS with Tween-20 (TBST), the secondary antibodies were added, finally assayed by enhanced chemiluminescence (ECL) Substrate. In related assays, β-actin (ab8226, Abcam, Cambridge, MA, USA) was used as the internal control.

### Transwell assays

HCC cells were planted on the top of 24-well Matrigel-coated transwell chambers at a density of 2 × 10^4^ cells per well. The lower chambers were loaded with complete medium. Twenty-four hours later, cells in the upper layer were removed and then fixed in methanol solution for 15 min. Crystal violet was adopted to stain the membranes for 10 min, and the invaded cells were observed and counted under a microscope (10 × 10).

### RNA immunoprecipitation (RIP) assay

With the Imprint RNA Immunoprecipitation Kit, RIP in Huh7 or MHCC97H cells was achieved with specific antibodies and normal control anti-IgG antibody (no. 3420, cell signaling, Boston, Massachusetts, USA). Lysates were obtained from HCC cell lines using RIP lysis buffer. The lysis was incubated with the magnetic beads conjugated with the AGO2 antibody (no. 2897, cell signaling, Boston, Massachusetts, USA) or IgG antibody (negative control). The precipitated RNAs went through RT–qPCR analysis.

### Dual luciferase reporter assay

For gene promoter analysis, CHMP4B promoter was subcloned into the pGL3-basic vector (E1751, Promega, Madison, WI, USA) and co-transfected with M1-Exo into Huh7 and MHCC97H cells. In addition, circFUT8-WT/Mut, CHMP4B-WT/Mut, and METTL14-WT/Mut vectors were respectively subcloned into the pmirGLO luciferase reporter vector (E1330, Promega, Madison, WI, USA) and then transfected with different plasmids in HCC cells. The Dual Luciferase Reporter Assay System (E1910, Promega, Madison, WI, USA) was used for the analysis.

### Flow cytometry

After 48 h transfection, HCC cells were collected and washed with PBS. They were double stained in a darkroom for 15 min, and then subjected to staining using the eBioscience Annexin V-FITC Apoptosis Detection Kit (85-BMS500FI-300, Invitrogen, Carlsbad, CA, USA). Finally, the apoptosis rate was analyzed through flow cytometry (BD Biosciences, Franklin Lake, New Jersey, USA).

### Subcellular fractionation detection

The PARIS kit (AM1921, Invitrogen, Carlsbad, CA, USA) was used for this assay, following the manufacturer’s instructions.The expression pattern of circFUT8, U3 (nucleus control), or ACT8 (cytoplasmic control) in two fractions was assessed by RT–qPCR. In related assays treated with M1-Exo, the percent of circFUT8 in the control or Exo group was determined by RT–qPCR.

### RNA pull down assay

The biotin-labeled circFUT8 probe with Bio-NC was obtained from RiboBio (Guangzhou, Guangdong, China) for the RNA pull down assay. Cells were lysed with lysis buffer, and then the lysates were incubated with specific biotin-labeled probes for 2 h. Then, the mixtures were incubated with the streptavidin beads to pull down the biotin-labeled RNA complex for another 4 h. After washing, the RNA complex was extracted with TRIzol and RNA enrichment was achieved through RT–qPCR.

### In vivo tumorigenesis experiment

Related in vivo studies were performed as previously described, with the approval of the Animal Care and Use Committee guidelines of our hospital (Date of the Ethic Committee decision: 25 January 2022) [20]. Male BALb/c nude mice (three mice per group) were bought from Guangdong Medical Laboratory Animal Center, and indicated HCC cells were subcutaneously injected into the flanks of nude mice at 2 × 10^6^ cells per site. Four weeks after injection, the mice were sacrificed, and tumors were removed to be weighed and measured.

### Statistical analysis

Each experiment was performed in triplicate. Data was exhibited as the mean ± standard deviation (SD). Statistical analyses were made in the form of Student’s *t* test (comparison for two groups) and one-way/two-way ANOVA (comparison for more than two groups). **P-values less than 0.01 (*P* < 0.01), representing the data that exhibited a significant statistical difference in the experimental group in comparison with the control.

## Results

### CircFUT8 expressed at a high level in HCC cells

**Fig. 1:**
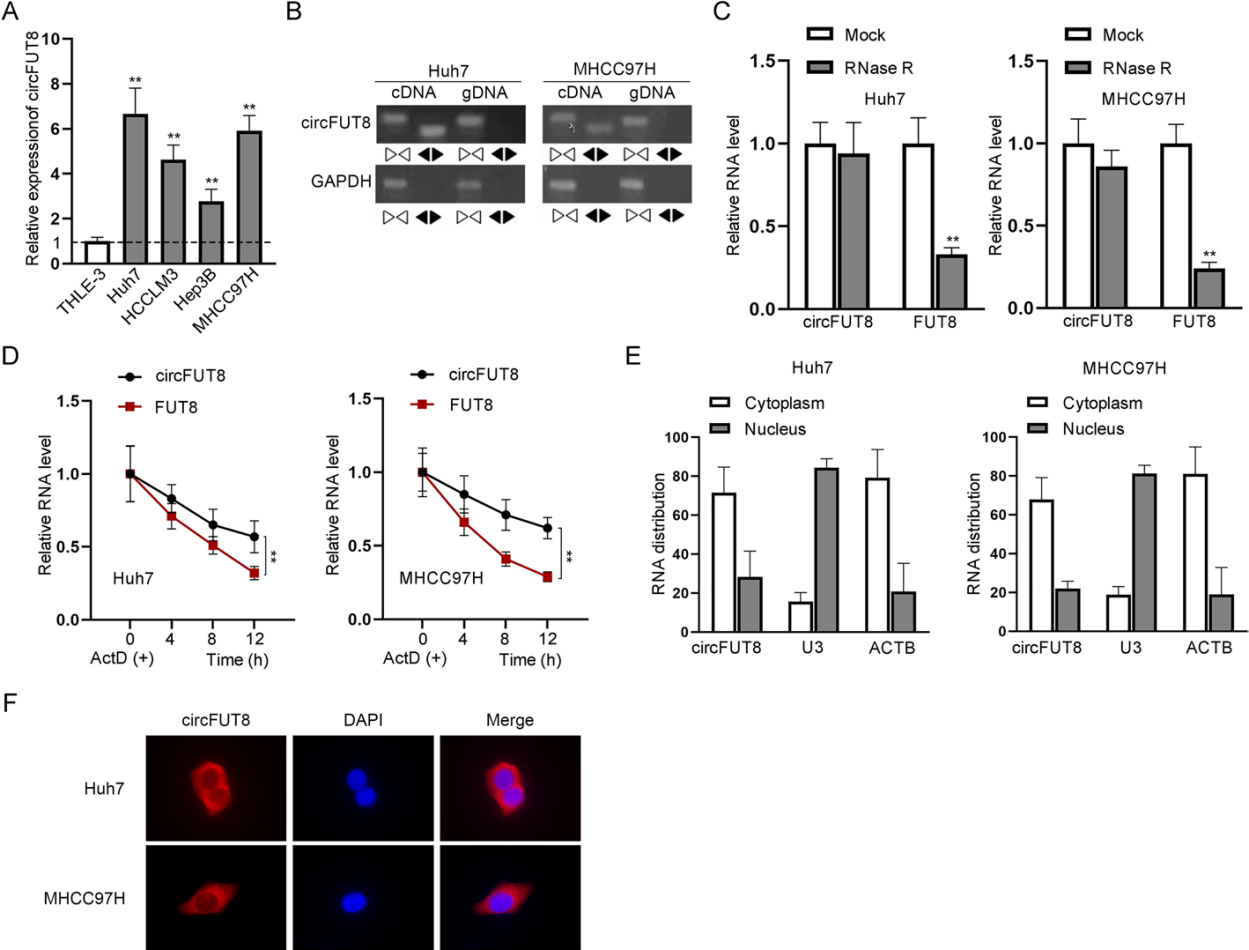
Confirmation of high circFUT8 expression in HCC cells. **A** CircFUT8 expression was tested in HCC cell lines (Huh7, HCCLM3, Hep3B, MHCC97H) compared with immortalized human liver epithelial THLE-3 cell line. **B**–**D** The circular structure of circFUT8 was confirmed through agarose gel electrophoresis and by treating HCC cells with Rnase R and Act D, respectively. **F** The identification of circFUT8 location in HCC cells was achieved by FISH. In **A**, **C**, **D** there was a significant statistical difference between the experimental group and the negative control group, with *P * < 0.01

RT–qPCR showed the higher expression of circFUT8 in HCC cell lines involving Huh7, HCCLM3, Hep3B, and MHCC97H in contrast to immortalized human liver epithelial cells THLE-3 (Fig. 1A), particularly in Huh7 and MHCC97H cells. CircFUT8 could only be amplified by the divergent primers in cDNA (Fig. 1B), and it was hardly changed following Rnase R treatment and the addition of Act D, which verified the circular structure of circFUT8 (Fig. 1C, D). As shown in subcellular fractionation and fluorescent in situ hybridization (FISH), the majority of circFUT8 was in the cytoplasm of HCC cells (Fig. 1E, F).

### CircFUT8 knockdown suppressed HCC cell malignancy

**Fig. 2:**
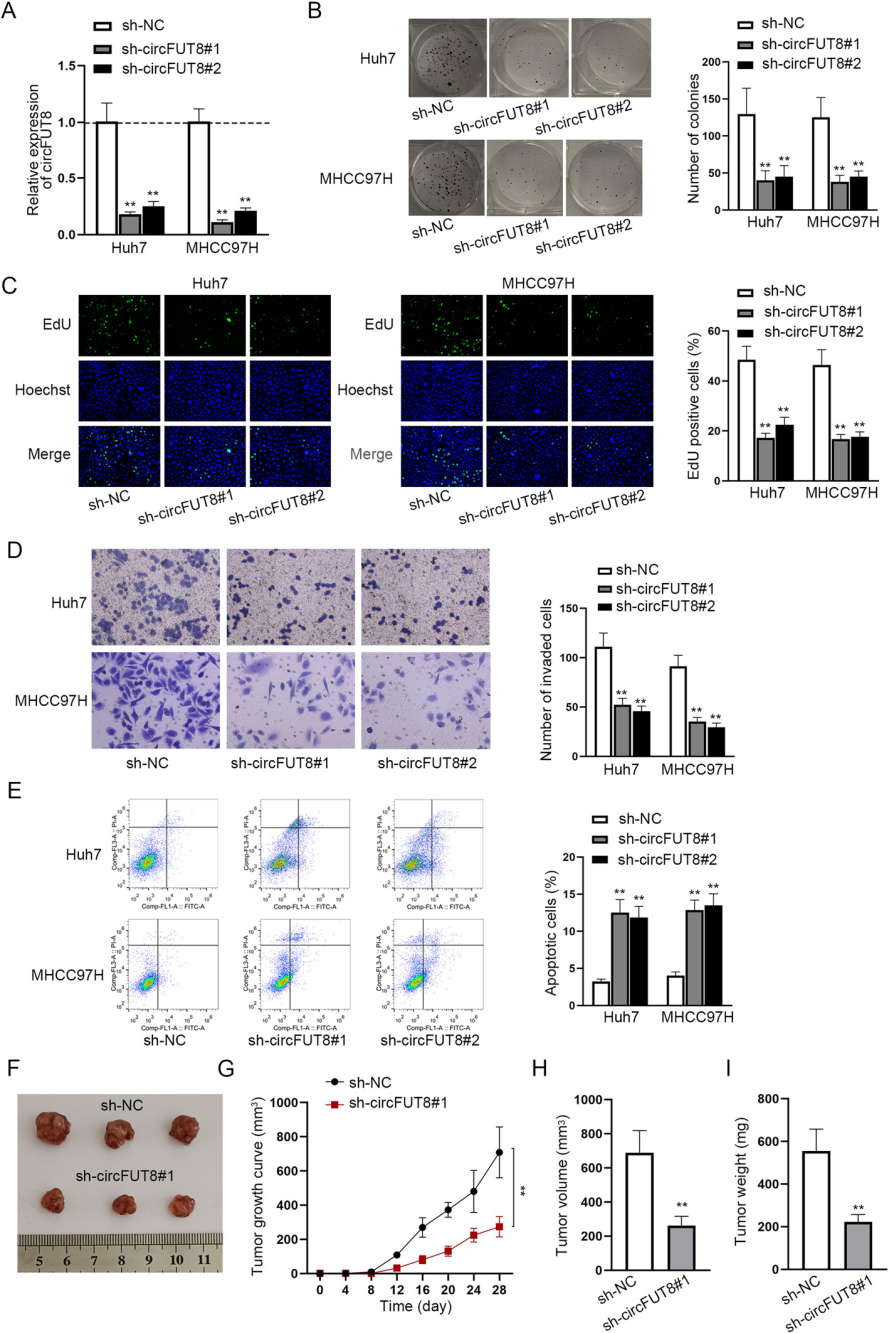
Inhibition of circFUT8 suppressed HCC cell malignancy and tumor growth. **A** CircFUT8 expression was reduced by sh-circFUT8#1/2 transfection in HCC cells. **B**, **C** Colony formation and EdU assays were conducted to verify the proliferation of HCC cells with the transfection of sh-circFUT8. **D** The invasion of HCC cells after circFUT8 depletion was evaluated by transwell assays. **E** The apoptosis rate of HCC cells after circFUT8 depletion was analyzed through flow cytometry. **F**–**I** In vivo tumor assays were performed to measure the tumor size, growth, volume, and weight after circFUT8 knockdown. The experimental group treated with sh-circFUT8#1/2 exhibited a significant statistical difference in comparison with the control sh-NC group, with a *P*-value < 0.01

By transfecting sh-circFUT8#1/2 into HCC cells, we obtained decreased circFUT8 expression, which produced a favorable outcome (Fig. 2A). Reduced colony numbers and less EdU-positive cells owing to circFUT8 silencing meant that circFUT8 inhibition alleviated cell proliferation (Fig. 2B, C). Also, invaded cells were reduced after circFUT8 inhibition, as shown by transwell assays in Fig. 2D. CircFUT8 inhibition promoted the apoptosis rate of HCC cells, as evidenced by flow cytometry (Fig. 2E). As observed in the animal studies, mice transfected with sh-circFUT8#1 exhibited smaller tumor size, with less tumor volume, and tumor weight (Fig. 2F–I). Therefore, we could draw the conclusion that circFUT8 inhibition suppressed tumor growth in vivo.

We overexpressed circFUT8 in Hep3B cells and further confirmed that circFUT8 overexpression could aggravate malignant cell behaviors and inhibit cell apoptosis (Additional file 1: Figure S1A–E).

### CircFUT8 competitively bound to miR-552-3p to elevate CHMP4B expression

**Fig. 3:**
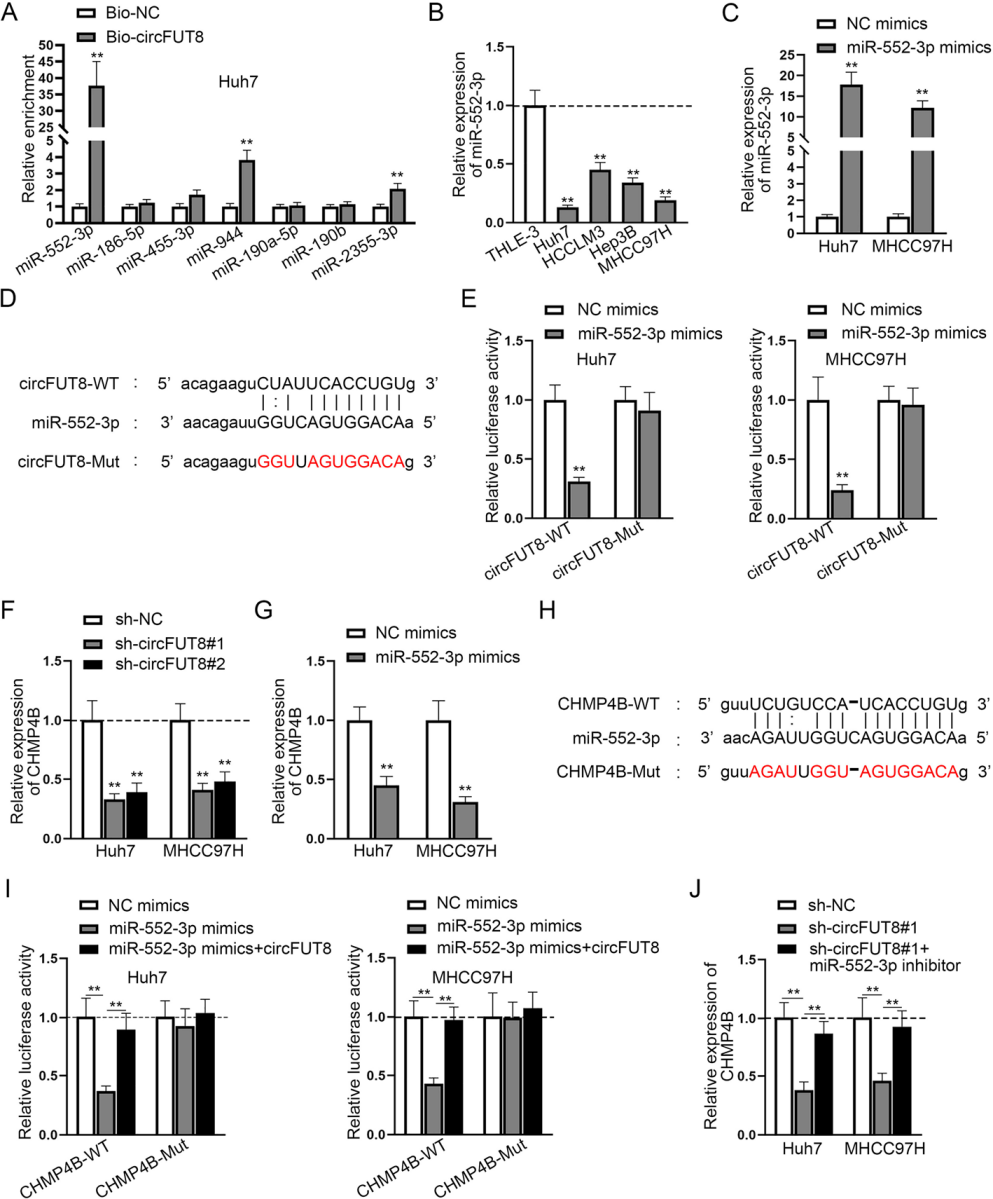
CircFUT8 sponged miR-552-3p to elevate CHMP4B expression. **A** Potential miRNAs of circFUT8 were forecast through starBase and the enrichment of selected miRNA candidates was measured in the bio-circFUT8 group via the RNA pull down assay. **B** MiR-552-3p expression in HCC cell lines relative to normal THLE-3 cells. **C** The favorable overexpression efficiency of miR-552-3p in HCC cells. **D** The binding sites between circFUT8 and miR-552-3p as well as the mutated sequences were predicted through starBase. **E** The luciferase activity of HCC cells transfected with miR-552-3p mimics and NC mimics in wild type and mutant circFUT8 vectors. **F**, **G** The influence of circFUT8 silencing or miR-552-3p overexpression on CHMP4B expression was assessed by luciferase reporter assay. **H** The binding sites and the mutated sequences between miR-552-3p and CHMP4B were forecasted through starBase. **I** The interaction among miR-552-3p, circFUT8, and CHMP4B in HCC was confirmed via luciferase reporter data. **J** CHMP4B expression in different groups was tested by RT–qPCR. In comparison with the negative control group, data in the experimental group was observed to be statistically significant (*P* < 0.01)

Combined with the subcellular distribution of circFUT8 determined in Fig. 1E, F, and the fact that circFUT8 can function as a competing endogenous RNA (ceRNA) [8], we then identified the downstream mechanism circFUT8 may exert on HCC progression. At first, we used starBase (http://starbase.sysu.edu.cn) and selected seven potential microRNAs (miRNAs) (selection condition: CLIP-Data >  = 5) that could bind to circFUT8: miR-552-3p, miR-186-5p, miR-455-3p, miR-944, miR-190a-5p, miR-190b, and miR-2355-3p. It was then shown that miR-552-3p was pulled down by the circFUT8 probe with the highest enrichment (Fig. 3A). Therefore, miR-552-3p was chosen for further investigations, and miR-552-3p expression was observed to be lower in HCC cells, particularly in Huh7 and MHCC97H cells (Fig. 3B). The favorable overexpression efficiency of miR-552-3p and the binding sites between circFUT8 and miR-552-3p were obtained using bioinformatics tools and RT–qPCR analysis (Fig. 3C, D). HCC cells displayed reduced luciferase activity after miR-552-3p mimics was transfected into the circFUT8-WT vector (Fig. 3E). After that, we sifted out charged multivesicular body protein 4B (CHMP4B) as the downstream molecule (selection condition: CLIP-Data >  = 5; Degradome-Data >  = 3; programNum >  = 4). RT–qPCR showed that circFUT8 knockdown or miR-552-3p overexpression inhibited CHMP4B expression in HCC cells (Fig. 3F, G). The binding sites and the mutated sequences between miR-552-3p and CHMP4B were also determined via starBase (Fig. 3H). Moreover, it was seen that the decreased luciferase activity caused by miR-552-3p mimics was then normalized by adding circFUT8, while the corresponding mutant group displayed no visible changes (Fig. 3I). Furthermore, circFUT8 depletion decreased CHMP4B expression, but knocking down miR-552-3p could normalize this impact (Fig. 3J). To summarize, circFUT8 could sponge miR-552-3p to elevate CHMP4B expression.

### M1-polarized macrophages inhibited HCC cell growth

Tumor-associated macrophages (TAMs) can be divided into M1-polarized macrophages and M2-polarized macrophages. Functionally speaking, M1 macrophages are tumor suppressors, while M2 macrophages promote cancer progression. As previously described [11], we used 150 nM PMA to induce incubated THP-1 cells into M0 macrophages, and then we used 20 ng/ml of IFN-γ and 10 ng/ml of LPS to treat M0 cells to form M1 macrophages (during 24 h). We could see that the expression of related macrophages markers (CD68/CD71/CD36) was upregulated after PMA treatment, which indicated the successful induction of THP-1 cells into M0 macrophages (Additional file 2: Fig. S2A). Furthermore, upon IFN-γ and LPS treatment, the expression of M1 macrophages-related markers [Tumor necrosis factor (TNF)-α, Interleukin (IL)-1β, IL-6, andC-X-C motif chemokine 10 (CXCL10)] all increased in M0 cells, indicating the successful induction of M1 macrophages (Additional file 2: Fig. S2B). Subsequently, a co-culture model of M1 macrophages and HCC cells was established (Additional file 2: Fig. S2C), and we could see that the proliferation of HCC cells was inhibited while cell apoptosis ability was enhanced upon co-transfection of M1 macrophages (Additional file 2: Fig. S2D–F). The above data suggested that M1 macrophages inhibited HCC cell growth.

### M1 macrophages-derived exosomes inhibited HCC cell growth

As exosomes are known as an important medium in the communication between cancer cells and noncancer cells, we speculated that M1 macrophages may reduce HCC malignancy by transferring exosomes into HCC cells. Therefore, we conducted related experiments to detect the existence of exosomes, trying to determine their effects on HCC cells. Through transmission electron microscopy, we observed the existence of M1-Exo and the expression of exosomes markers (CD63/CD81) in the exosomes (Additional file 3: Fig. S3A, B). Also, PKH67-marked exosomes were sponged by HCC cells (Additional file 3: Fig. S3C). Moreover, after HCC cells were treated with M1-Exo, we could see that their proliferative abilities were hindered while the apoptosis rate was promoted (Additional file 3: Fig. S3D–F). To conclude, exosomes derived from M1-polarized macrophages inhibited HCC cell growth.

### M1 macrophages-derived exosomes inhibited the nuclear export of circFUT8

**Fig. 4:**
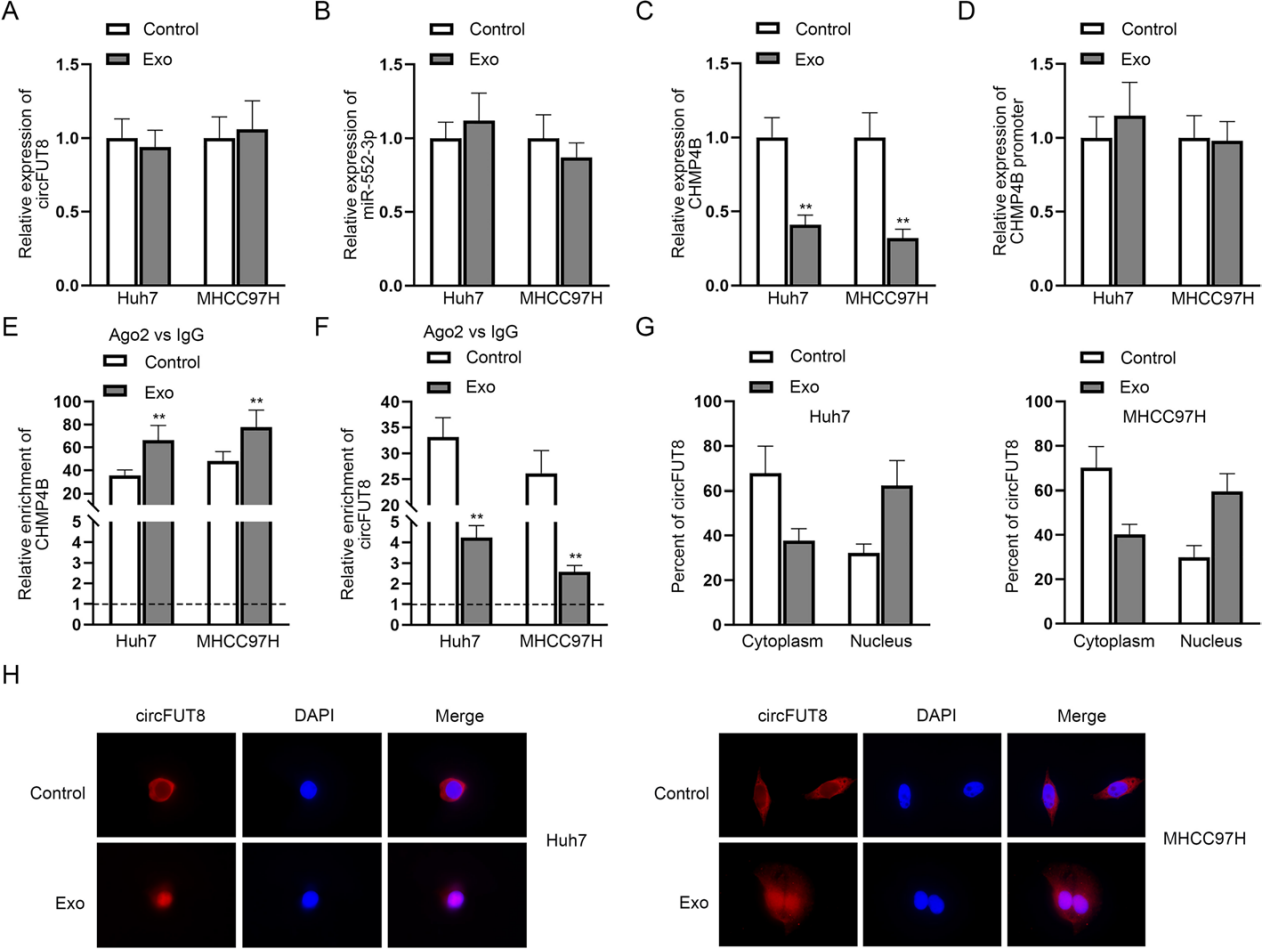
M1 macrophages-derived exosomes inhibited the nuclear export of circFUT8. **A**–**C** CircFUT8, miR-552-3p, and CHMP4B expression in HCC cells treated with M1-Exo or control was tested via RT–qPCR. **D** The effect of M1-Exo on the luciferase activity of CHMP4B promoter region in HCC cells. **E**, **F** RIP assay was adopted to assess CHMP4B and circFUT8 enrichment after HCC cells were treated with M1-Exo. **G**, **H** CircFUT8 distribution in HCC cells treated with M1-Exo or control was determined by nucleus–cytoplasmic separation and FISH assays. In **C** and **B**, data in the experimental group had statistical significance, with *P* < 0.01

In this experiment, we tried to verify whether M1-Exo may affect the circFUT8/miR-552-3p/CHMP4B pathway. RT–qPCR demonstrated that M1-Exo did not affect circFUT8 or miR-552-3p expression, while it obviously reduced CHMP4B expression in HCC cells (Fig. 4A–C). M1-Exo exerted no influence on the activity of the CHMP4B promoter region, which meant M1-Exo did not affect CHMP4B transcription (Fig. 4D). Therefore, we speculated that M1-Exo may exert post-transcriptional effects on CHMP4B expression, so we then focused on examining whether M1-Exo may affect the formation of the ceRNA network involving circFUT8/miR-552-3p/CHMP4B at the post-transcriptional level. M1-Exo significantly increased the enrichment of CHMP4B in Ago2-RISC, which may be caused by the significantly downregulated enrichment of circFUT8 (Fig. 4E, F). Since cytoplasmic circRNAs play a major role in the ceRNA pattern, while M1-Exo barely changed the total circFUT8 expression, as shown in Fig. 4A, we speculated that M1-Exo may affect the distribution of circFUT8 in HCC cells and reduce the cytoplasmic level of circFUT8, thereby inhibiting the above ceRNA mechanism. Therefore, we conducted nucleus–cytoplasmic separation and FISH assays to detect cell distribution changes of circFUT8 in HCC cells (Fig. 4G, H), which showed that circFUT8 expression was decreased in the cytoplasm while increased in the nucleus. Therefore, we concluded that M1-Exo inhibited the nuclear export of circFUT8.

### M1 macrophages-derived exosomes reduced the m6A level of circFUT8 by inhibiting METTL14

**Fig. 5:**
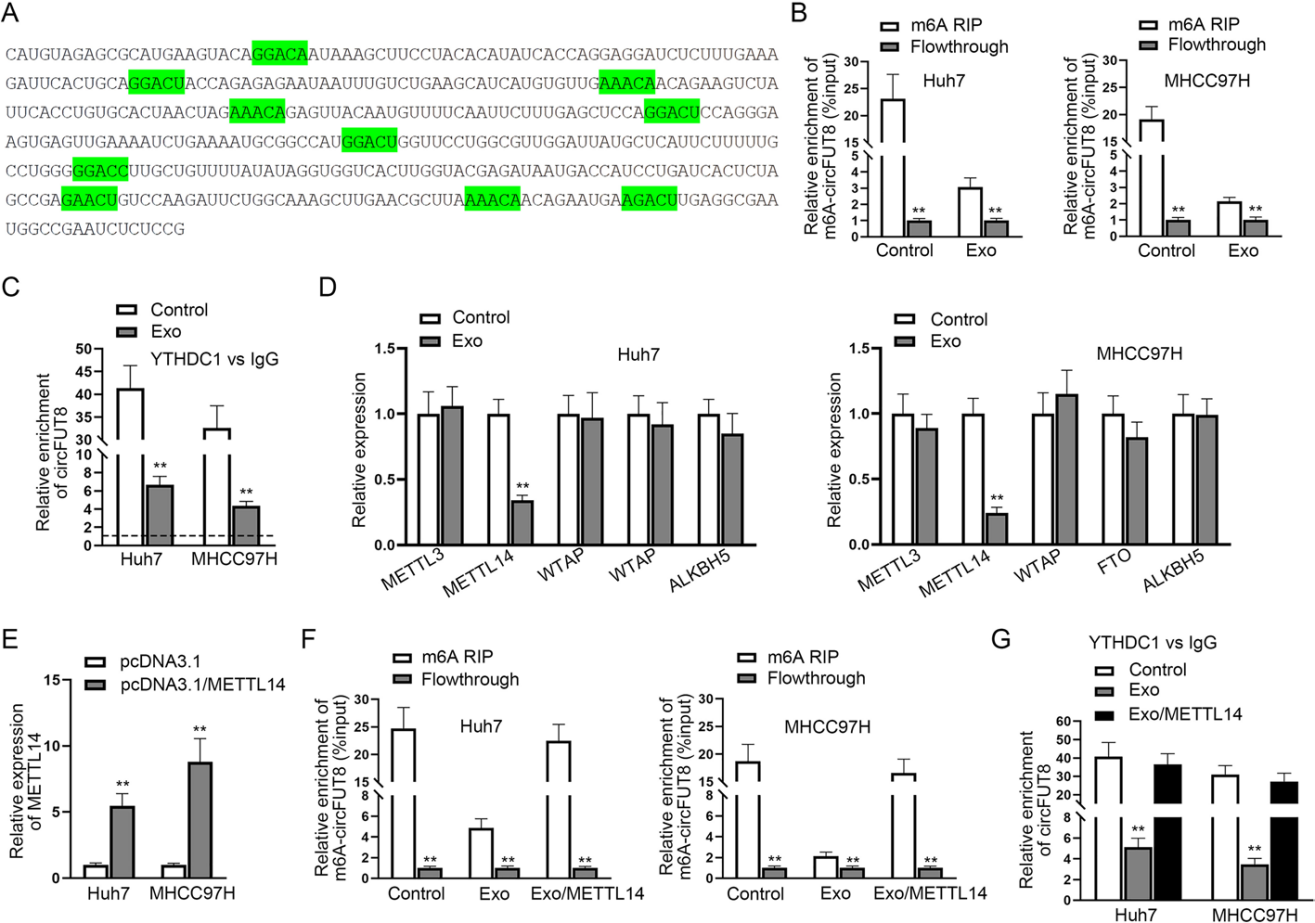
M1 macrophages-derived exosomes reduced the m6A level of circFUT8 by inhibiting METTL14. **A** The possible m6A modification sites in the circFUT8 sequence. **B** The effect of M1-Exo on the m6A-modified circFUT8 in HCC cells was analyzed through the MeRIP assay. **C** RIP data of the enrichment of circFUT8 binding to YTHDC1 (the nuclear m6A reader) after adding M1-Exo in HCC cells. **D** The expression of five proteins that can modify RNA m6A (METTL3, METTL14, WTAP, FTO, and ALKBH5) was tested. **E** METTL14 expression was enhanced by pcDNA3.1/METTL14 transfection. **F** The effect of METTL14 overexpression on m6A-circFUT8 enrichment upon M1-Exo treatment was tested through the Me-RIP assay. **G** The interaction between YTHDC1 and circFUT8 upon M1-Exo treatment was determined through the RIP assay. In relevant assays, data in the experimental group was observed to be statistically significant compared with the negative control group (*P* < 0.01)

It has recently been determined that m6A modification exerts vital biological functions in regulating circRNA [21], and m6A-modified circRNAs are more prone to cytoplasmic export [19]. Therefore, we speculated that M1-Exo may affect the subcellular distribution of circFUT8 via m6A modification. First of all, we searched for the possible m6A modification sites in circFUT8 sequence, and the results are shown with green highlights (Fig. 5A). The enrichment of m6A-modified circFUT8 was significantly reduced after M1-Exo treatment (Fig. 5B). It was then observed from the RIP assay that after adding M1-Exo in HCC cells, the enrichment of circFUT8 binding to YTHDC1 (the nuclear m6A reader) was significantly reduced (Fig. 5C). The above data demonstrated that M1-Exo inhibited m6A modification of circFUT8 in HCC cells. As previously described [22], METTL3, METTL14, WTAP, FTO, and ALKBH5 are five proteins that can affect the m6A modification, so we continued to explore whether M1-Exo may affect which specific factor effects circFUT8 m6A modification. RT–qPCR showed that among these five candidates, only METTL14 reduced M1-Exo treatment (Fig. 5D). Therefore, METTL14 was chosen for further investigation. The overexpression efficiency of METTL14 was tested, and then the MeRIP assay determined that overexpression of METTL14 countervailed the decreased m6A-circFUT8 enrichment after M1-Exo treatment (Fig. 5E, F). It was further shown from the RIP assay that the enrichment of circFUT8 binding to YTHDC1 was suppressed by M1-Exo, while overexpressing METTL14 could countervail this effect (Fig. 5G). The above data indicated that M1-Exo inhibited the m6A modification of circFUT8 by inhibiting METTL14 in HCC cells.

### M1-Exo transferred miR-628-5p to inhibit METTL14 expression in HCC

**Fig. 6:**
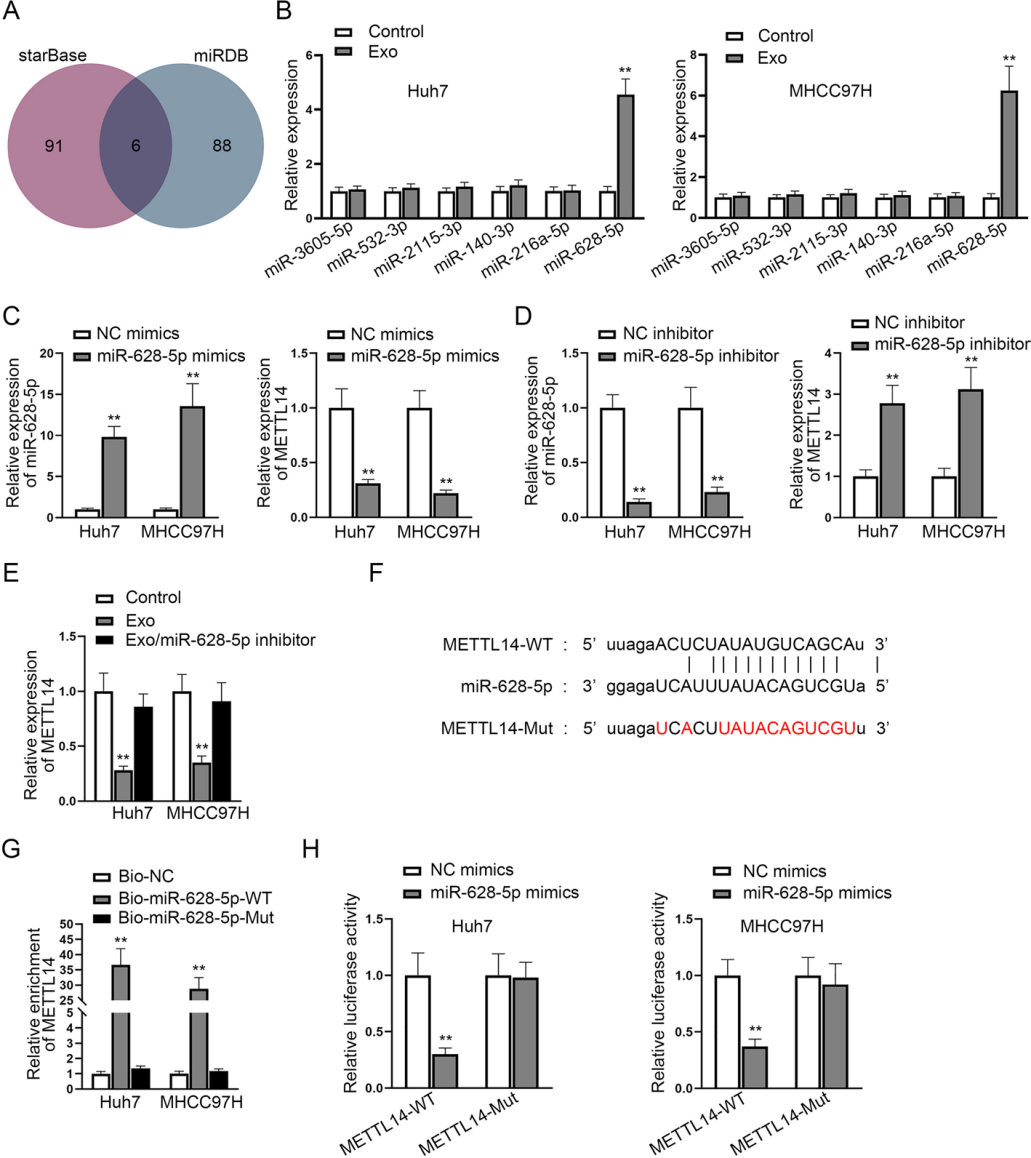
M1-Exo transferred miR-628-5p to inhibit METTL14 expression in HCC. **A** Potential miRNAs in the upstream of METTL14 were selected through bioinformatic predictions. **B** The expression of potential miRNAs following M1-Exo treatment was tested. **C**, **D** MiR-628-5p expression was enhanced or silenced in HCC cells, and then the expression of METTL14 was analyzed by RT–qPCR. **E** METTL14 expression was measured under different conditions. **F** The binding sites between METTL14 and miR-628-5p were forecast through starBase. **G** An RNA pull down assay was conducted to evaluate the enrichment of METTL14 in wild type or mutated bio-miR-628-5p groups. **H** The luciferase activity of HCC cells transfected with miR-628-5p mimics in the wild type or mutated METTL14 groups. In relevant assays, data in the experimental group was statistically significant in comparison with the negative control group (*P* value < 0.01)

MiRNAs are common upstream inhibitors of protein-coding genes. Therefore, we tried to explore whether M1-Exo may inhibit METTL14 expression in HCC cells by transferring certain miRNAs. We first applied starBase and miRDB (http://mirdb.org/) databases to forecast potential miRNAs in the upstream of METTL14, and six common miRNAs were presented in the intersection (Fig. 6A). RT–qPCR showed that only miR-628-5p expression was enhanced after the treatment of M1-Exo in HCC cells (Fig. 6B), and that miR-628-5p negatively regulated METTL14 expression in HCC cells (Fig. 6C, D). Moreover, it was observed that METTL14 expression was reduced by M1-Exo, but such effect was countervailed by the addition of miR-628-5p inhibitor (Fig. 6E). The binding sites between METTL14 and miR-628-5p are shown in Fig. 6F. METTL14 enrichment was enhanced in the wild-type miR-628-5p probe (Fig. 6G). Furthermore, the luciferase reporter assay determined that the transfection of miR-628-5p mimics inhibited the luciferase activity of the METTL14-Wt group rather than the mutant group (Fig. 6H). In summary, M1-Exo transferred miR-628-5p to inhibit METTL14 expression in HCC cells.

### M1-Exo regulated the circFUT8/CHMP4B axis via METTL14 to inhibit HCC cell progression

**Fig. 7:**
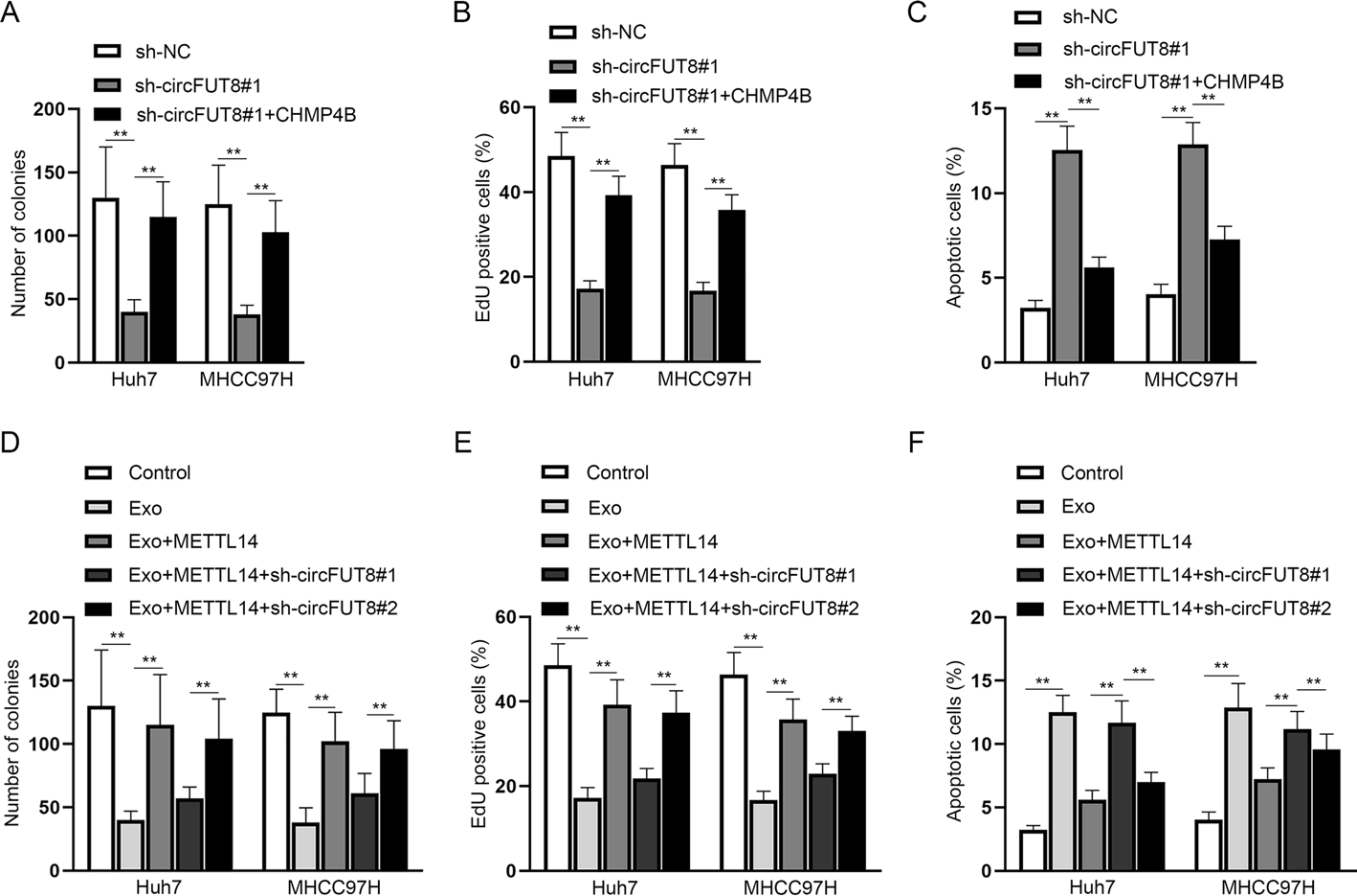
M1-Exo regulated the circFUT8/CHMP4B axis via METTL14 to inhibit HCC cell progression. **A**–**C** The proliferation and apoptosis of HCC cells in different groups transfected with sh-NC, sh-circFUT8#1, and sh-circFUT8#1 + CHMP4B plasmids was verified through colony formation together with EdU and flow cytometry assays. **D**–**F** HCC malignant cell behaviors were evaluated through functional assays in different groups (control, Exo, Exo + METTL14, Exo + METTL14 + sh-circFUT8#1, and Exo + METTL14 + sh-circFUT8#2). In relevant assays, data in the experimental group was statistically significant in comparison with the negative control group (*P* < 0.01)

Data from the colony formation assay together with the EdU assay showed that circFUT8 silencing inhibited the proliferation of HCC cells, while CHMP4B overexpression recovered this impact (Fig. 7A, B). Also, the upregulation of CHMP4B countervailed the enhanced HCC cell apoptosis caused by circFUT8 silencing (Fig. 7C). The above finding indicated that circFUT8 promoted HCC cell progression via CHMP4B. Furthermore, it was unveiled through functional assays that the addition of M1-Exo inhibited cell proliferation and induced cell apoptosis in HCC, while METTL14 overexpression countervailed such impacts. On this basis, inhibiting circFUT8 expression again normalized the above rescue effects in HCC cells (Fig. 7D–F). The above data disclosed that M1-Exo inhibited METTL14 to further inhibit the circFUT8/CHMP4B axis, thereby repressing HCC cell malignancy.

## Discussion

Known as one of the most prevalent cancers in the world, HCC is frequently diagnosed at an advanced stage, which is a challenge for research into the improvement of HCC early diagnosis and treatments [22]. CircFUT8 has been shown to be both a tumor suppressor and an oncogene in the development of cancers [8, 9], but it has not been studied in HCC. In the current study, we proved the oncogenic property of circFUT8 in regulating HCC cell malignancy, and its aggravation of tumor growth was also determined in mice. Through the identification of circFUT8 location in HCC cells, we found that circFUT8 was distributed in the cytoplasm of HCC cells. Cytoplasmic circRNAs have been proven to be miRNA sponges in cancer [23]. Consistent with this report, we verified that circFUT8 competitively bound to miR-552-3p to increase CHMP4B expression, which constitutes a new ceRNA pathway in HCC cells. MiR-552-3p is an effective target in inhibiting cell growth [24] and CHMP4B can be regarded as a promising prognostic biomarker for HCC [25].

Tumor-associated macrophages play an important role in the inflammatory microenvironment, and thus contributing to HCC progress via M1 and M2 polarization. Among them, classically activated M1 macrophages repress tumor growth by releasing pro-inflammatory factors [26, 27]. Exosomes are membrane-encased vesicles derived by nearly all cell types for intercellular communication as well as regulation, and it has been reported that M1-exosomes can act as a drug carrier to facilitate the antitumor effects of chemotherapeutics in tumor-bearing mice [28]. In our current study, a co-culture model of M1 macrophages and HCC cells was constructed, and it was discovered that both M1 macrophages and exosomes derived from them exerted an inhibitory impact on HCC cell growth.

The link between m6A modification and cancer development has been well elucidated [29], and related studies have also uncovered that m6A modification of circRNA increases export to the cytoplasm [19]. During our investigations, it was unveiled that M1-Exo transferred miR-628-5p to HCC cells to inhibit METTL14 expression. Moreover, METTL14 was found to promote the m6A modification of circFUT8 so as to promote its nuclear export to the cytoplasm. METTL14 has been reported to mediate the m6A modification in the modulation of colorectal cancer [30], breast cancer [31], and HCC [32].

As illustrated by the graphical abstract, HCC cells could absorb the exosomal-miR-628-5p delivered by M1 macrophages, which increased miR-628-5p expression and thus inhibited METTL14 in HCC cells. In the nucleus, METTL14 participated in the m6A modification of circFUT8, which led to the recognition of YTHDC1 protein and the transfer of circFUT8 to the cytoplasm. Meanwhile, cytoplasmic circFUT8 competitively bound to miR-552-3p to upregulate CHMP4B mRNA, which finally accelerated the malignant cell behaviors in HCC.

## Conclusions

We demonstrated the oncogenic role of circFUT8 in HCC, and exosomal miR-628-5p derived from M1 macrophages could inhibit the m6A modification of circFUT8 so as to suppress HCC progression. We are the first to verify the interactions among circFUT8, M1 macrophages, exosomes, and m6A modification in HCC development. Though more studies are needed to verify the clinical and prognostic values of circFUT8, it is our hope that our study can help offer fresh guidance for the theoretical understanding of HCC mechanism, as well as for the exploration of effective biomarkers for HCC treatment.

## Supplementary Information

**Additional file 1: Figure S1. **The overexpression of circFUT8 promoted HCC cell malignancy. (A) CircFUT8 expression was elevated in Hep3B cells. (B-E) The aggravation of circFUT8 overexpression on HCC cell malignancy was assessed via functional assays. Data in the experimental group treated with elevated circFUT8 expression was observed to be with statistical significance in comparison with the negative control group (p value < 0.01).

**Additional file 2: Figure S2. **M1 polarized macrophages inhibited HCC cell proliferation. (A) The expression of related macrophages markers (CD68/CD71/CD36) after PMA treatment in THP-1 cells was tested. (B) The expression of M1 macrophages related markers in M0 cells after the treatment of IFN-γ and LPS. (C) The co-culture model of M1 macrophages and HCC cells. (D-F) The influence of M1 co-transfection on HCC cell proliferation and apoptosis was confirmed through functional assays. In relevant assays, data in the experimental group was observed to be with statistical significance in comparison with the negative control group (p value < 0.01).

**Additional file 3: Figure S3. **M1 macrophages-derived exosomes inhibited HCC cell proliferation. (A) Transmission electron microscopy was applied for the observation of M1 macrophages-derived exosomes. (B) Western blot analysis on exosomes markers CD63/CD81 expression. (C) PKH67 Green Fluorescent Cell Linker Kit was applied to detect the absorption of PKH67-marked exosomes by HCC cells. (D-F) Functional assays were carried out to evaluate the effects of M1-Exo on the proliferation as well as apoptosis of HCC cells. In relevant assays, data in the experimental group was observed to be with statistical significance in comparison with the negative control group (p value < 0.01).

**Additional file 4. **Original images of agarose gel electrophoresis assay and western blot analysis.

## Data Availability

Not applicable.

## Abbreviations

circRNA: Circular RNA
miRNA: MicroRNA
mRNA: Messenger RNA
shRNA: Short hairpin RNA
ceRNA: Competing endogenous RNA
NC: Negative control
GAPDH: Glyceraldehyde-3-phosphate dehydrogenase
SD: Standard deviation
RT–qPCR: Quantitative real-time polymerase chain reaction
RIP: RNA-binding protein immunoprecipitation
RISC: RNA-induced silencing complex
EdU: 5-Ethynyl-2′-deoxyuridine
FISH: Fluorescent in situ hybridization
