# Supplementary figures and images for: Exosomal miR-628-5p from M1 polarized macrophages hinders m6A modification of circFUT8 to suppress hepatocellular carcinoma progression
Source: Cell Mol Biol Lett. 2022 Dec 6;27:106. doi: 10.1186/s11658-022-00406-9 (PMC9724320; doi:10.1186/s11658-022-00406-9)

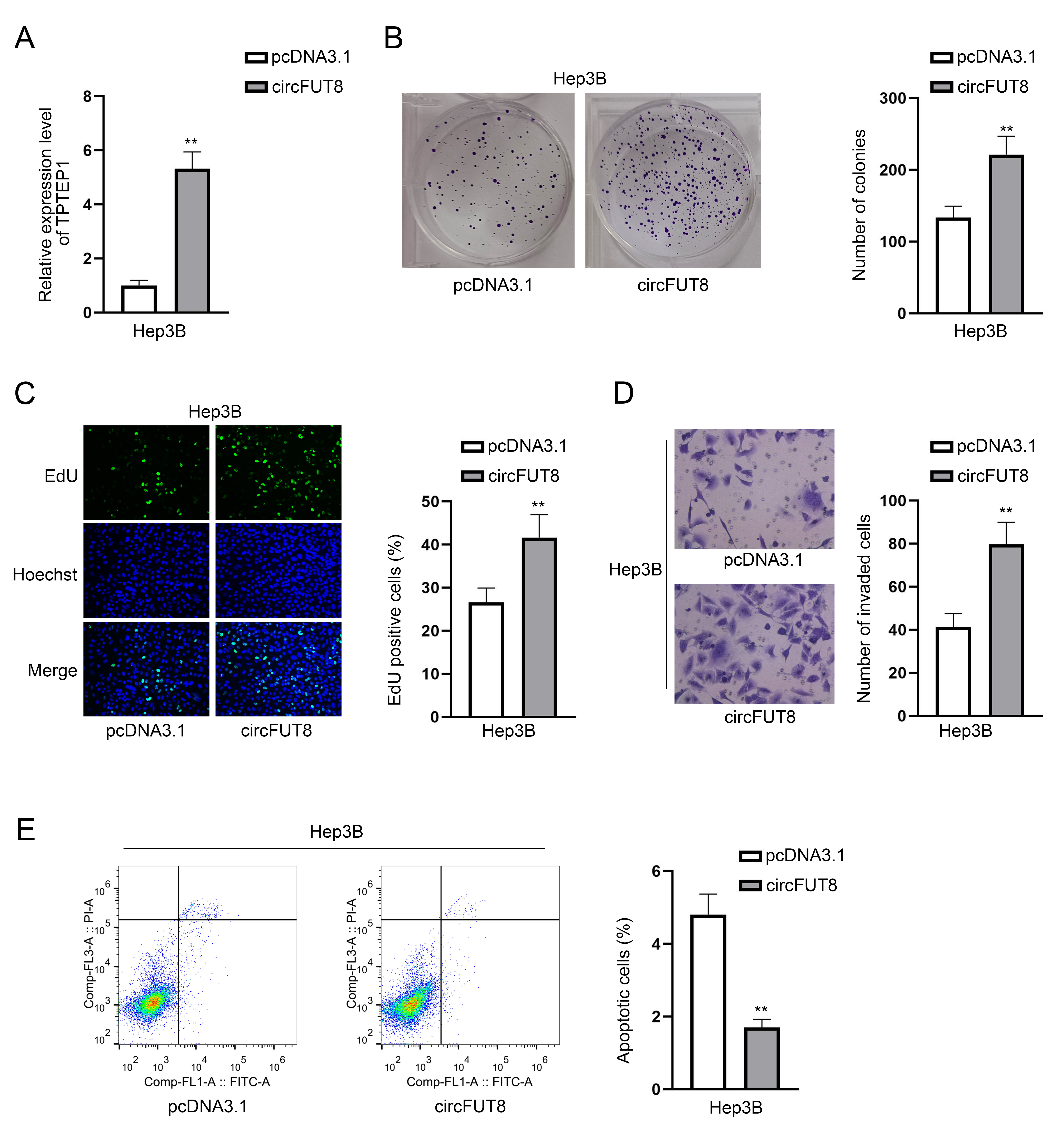

Supplement: Supplementary file 1 — Additional file 1: Figure S1. The overexpression of circFUT8 promoted HCC cell malignancy. (A) CircFUT8 expression was elevated in Hep3B cells. (B-E) The aggravation of circFUT8 overexpression on HCC cell malignancy was assessed via functional assays. Data in the experimental group treated with elevated circFUT8 expression was observed to be with statistical significance in comparison with the negative control group (p value < 0.01). [file 11658_2022_406_MOESM1_ESM.jpg]

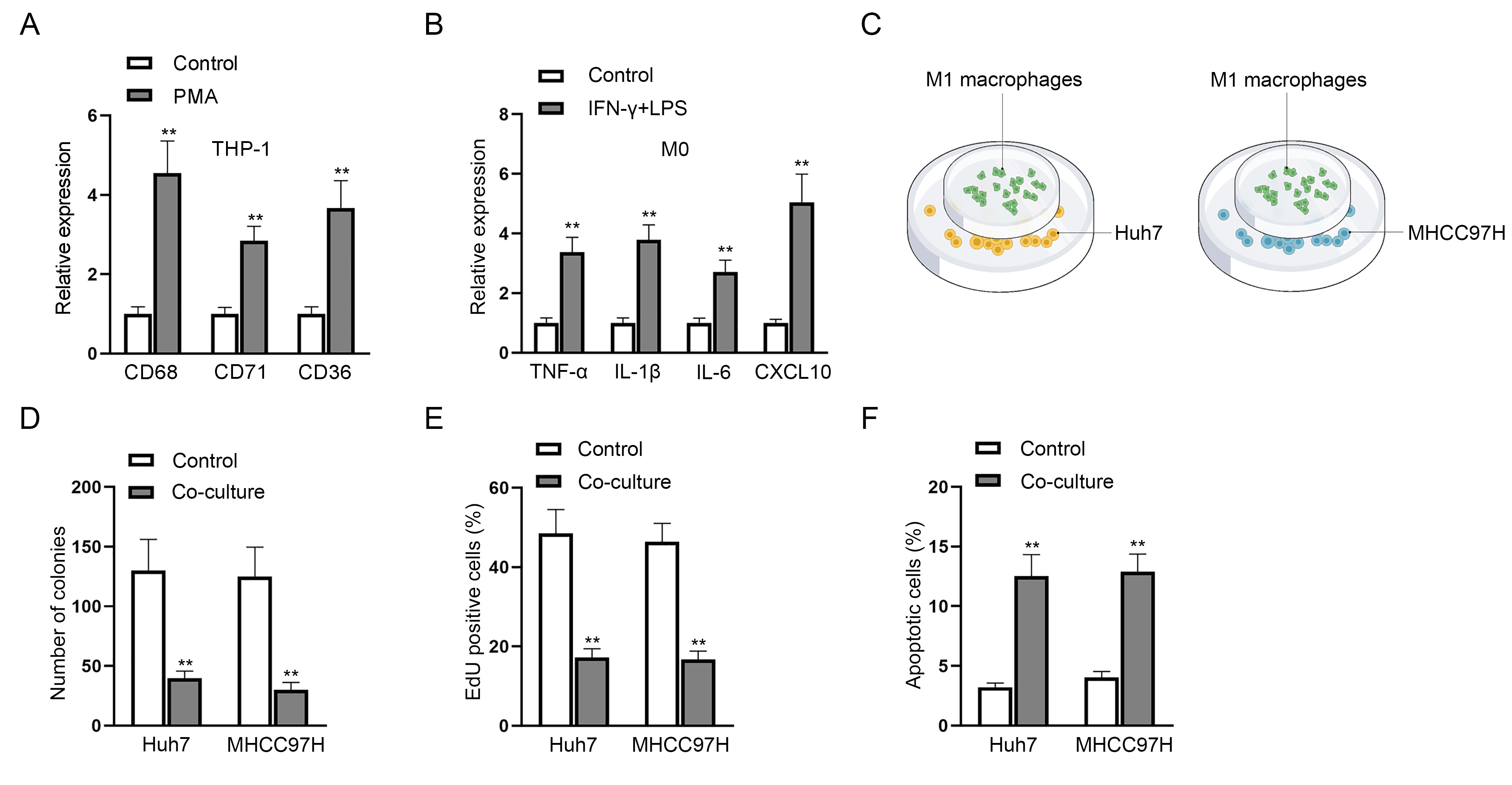

Supplement: Supplementary file 2 — Additional file 2: Figure S2. M1 polarized macrophages inhibited HCC cell proliferation. (A) The expression of related macrophages markers (CD68/CD71/CD36) after PMA treatment in THP-1 cells was tested. (B) The expression of M1 macrophages related markers in M0 cells after the treatment of IFN-γ and LPS. (C) The co-culture model of M1 macrophages and HCC cells. (D-F) The influence of M1 co-transfection on HCC cell proliferation and apoptosis was confirmed through functional assays. In relevant assays, data in the experimental group was observed to be with statistical significance in comparison with the negative control group (p value < 0.01). [file 11658_2022_406_MOESM2_ESM.jpg]

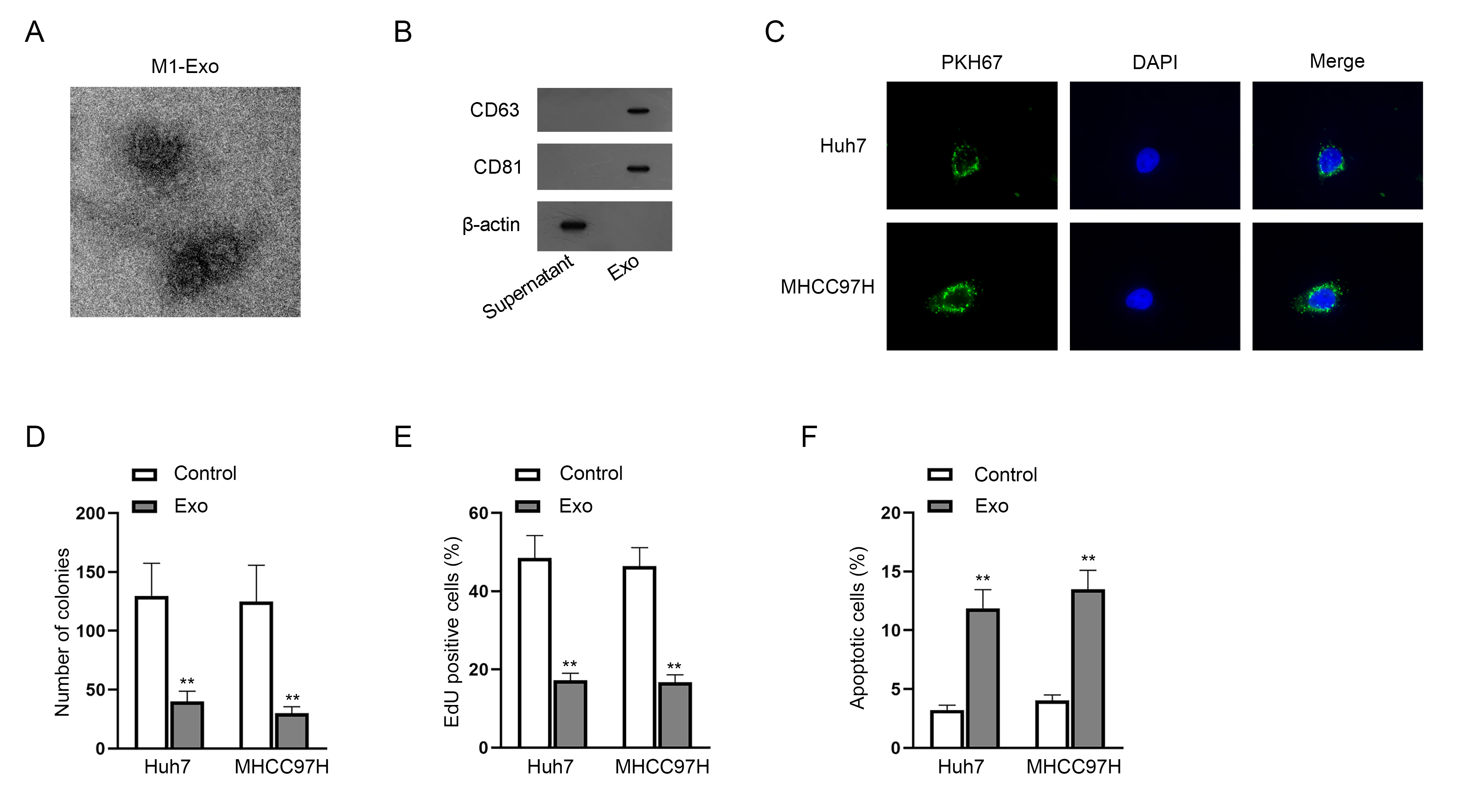

Supplement: Supplementary file 3 — Additional file 3: Figure S3. M1 macrophages-derived exosomes inhibited HCC cell proliferation. (A) Transmission electron microscopy was applied for the observation of M1 macrophages-derived exosomes. (B) Western blot analysis on exosomes markers CD63/CD81 expression. (C) PKH67 Green Fluorescent Cell Linker Kit was applied to detect the absorption of PKH67-marked exosomes by HCC cells. (D-F) Functional assays were carried out to evaluate the effects of M1-Exo on the proliferation as well as apoptosis of HCC cells. In relevant assays, data in the experimental group was observed to be with statistical significance in comparison with the negative control group (p value < 0.01). [file 11658_2022_406_MOESM3_ESM.jpg]

A

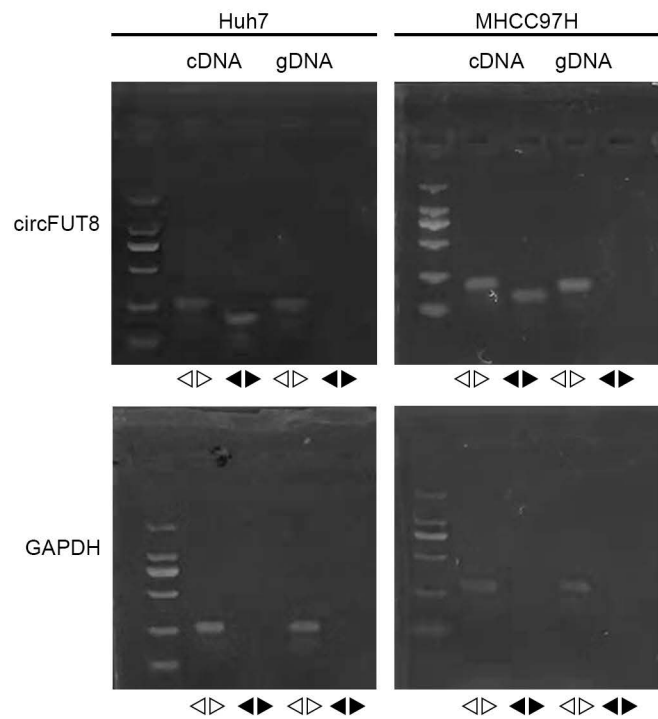

B

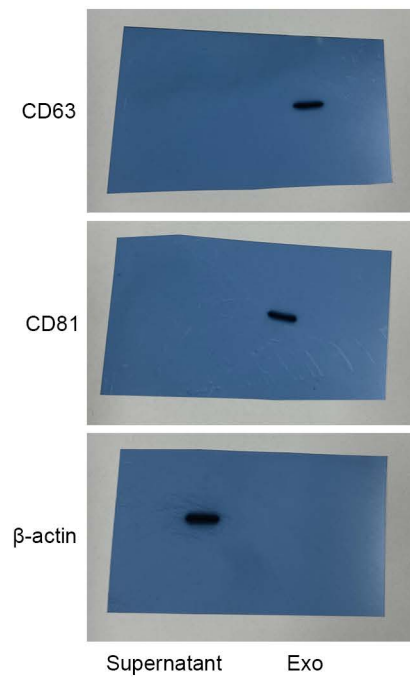

Supplement: Supplementary file 4 — Additional file 4. Original images of agarose gel electrophoresis assay and western blot analysis. [file 11658_2022_406_MOESM4_ESM.pdf]
